# Association Between Dietary Phytochemical Index and Neonatal Thyroid Function

**DOI:** 10.1155/2024/9558023

**Published:** 2024-05-28

**Authors:** Vida Hashemi Dehkordi, Mehri Khoshhali, Motahar Heidari-Beni, Elham Hashemi Dehkordi, Mahin Hashemipour, Neda Mostofizadeh, Seyede Shahrbanoo Daniali, Roya Kelishadi

**Affiliations:** ^1^ Department of Pediatric Endocrinology Endocrine and Metabolism Research Center Isfahan University of Medical Sciences, Isfahan, Iran; ^2^ Department of Pediatrics Child Growth and Development Research Center Research Institute for Primordial Prevention of Non-Communicable Disease Isfahan University of Medical Sciences, Isfahan, Iran; ^3^ Department of Nutrition Child Growth and Development Research Center Research Institute for Primordial Prevention of Non-Communicable Disease Isfahan University of Medical Sciences, Isfahan, Iran; ^4^ Department of Pediatric Endocrinology Child Growth and Development Research Centre Research Institute for Primordial Prevention of Non-Communicable Disease Isfahan University of Medical Sciences, Isfahan, Iran; ^5^ Metabolic Liver Diseases Research Center Imam Hossein Children's Hospital Isfahan University of Medical Sciences, Isfahan, Iran

**Keywords:** cord blood, phytochemicals, pregnancy, thyroid function

## Abstract

**Background:** Thyroid hormones regulate fetal growth and differentiation of several tissues. Maternal dietary patterns may be correlated with changes in the level of neonatal thyroid-stimulating hormone (TSH). We hypothesized that since maternal nutrition affects birth weight and offspring growth, it may also impact endocrine patterns in offspring. This study is aimed at assessing the relationship between maternal dietary phytochemical index (DPI) in the first trimester of pregnancy and neonatal cord blood thyroid hormone levels.

**Methods:** This cross-sectional study is a substudy of a birth cohort. Overall, 216 mothers, aged 16–45 years, were recruited in their first trimester of pregnancy. To calculate DPI, the daily energy percentage of phytochemical-rich foods was divided by the total daily energy intake. At delivery time, TSH and free thyroxine (FT4) levels were measured in cord blood samples using chemiluminescence immunoassay.

**Results:** The mean (standard deviation (SD)) age of mothers was 29.56 (5.50) years, and 47% of newborns were girls. The mean (SD) of DPI in the first, second, third, and fourth quartiles was 25.03 ± 4.67, 33.87 ± 2.18, 40.64 ± 2.10, and 51.17 ± 4.98, respectively. There was not any significant correlation between DPI score with cord serum TSH and FT4 levels in crude and adjusted analysis.

**Conclusion:** No significant relationship between maternal DPI with cord serum TSH and FT4 levels was shown. Limited experience exists about the effect of maternal diet quality indices on neonatal thyroid function, and further studies are needed in this regard.

## 1. Introduction

Thyroid hormones regulate the growth of fetal and differentiation of several tissues, such as brain, adipose tissue, and bone. The physiology of thyroid significantly changes during pregnancy and provides adequate hormones for the mother and fetus. During early pregnancy, the growth and development of fetus depend on the maternal thyroxine because the fetal thyroid gland is entirely mature after 20 weeks of gestation [1].

Several factors including maternal thyroid diseases, some drugs, type of delivery, dietary pattern, and birth conditions are associated with changes in the neonatal thyroid-stimulating hormone (TSH) concentration. Diet is one of the lifestyle factors that influence the risk of thyroid disorders [2, 3]. The neonatal thyroid is very sensitive to variations in maternal dietary intake. Neonatal TSH levels could show the intake of some nutrients including iodine during pregnancy. A healthy diet includes fruits, vegetables, nuts, and fish and contains useful micronutrients that are important for thyroid function [4]. These findings showed the importance of maternal diet during pregnancy on neonatal TSH levels [5, 6]. Proper nutrition and healthy dietary pattern may improve maternal thyroid function. Studies showed that fruits, vegetables, nuts, legumes, soya, whole grains, and olive oil that contain phytochemical compounds could reduce the risk of thyroid disorders [7, 8]. An easy and practical method for investigation of phytochemical intake is the calculation of dietary phytochemical index (DPI). DPI is defined as daily energy percentage of phytochemical-rich foods [9, 10].

The thyroid health of neonates has a significant effect on brain development. Maternal and infant factors affect infant TSH and thyroid hormone levels. However, conflicting findings have been reported [11].

We hypothesized that since maternal nutrition affects birth weight and offspring growth, it may also impact endocrine patterns in offspring. There is some evidence related to single nutrients and thyroid function. However, there is few evidence related to specific dietary patterns and thyroid function. In fact, no studies have yet assessed the possible impact of maternal DPI on neonatal thyroid function. Thus, this study is aimed at investigating the association between maternal DPI on neonatal cord blood thyroid hormone levels.

## 2. Methods

### 2.1. Study Design and Participants

This cross-sectional study is a substudy of the Prospective Epidemiological Research Studies in IrAN (PERSIAN) Birth Cohort in Isfahan Center titled “Isfahan Birth Cohort” (IBC). The study information was published previously [12].

Considering a significance level of 5% (*z* = 1.96), statistical power of 80% (*z* = 0.84), TSH variance of 4.84 [13], and *d* = 0.59, the sample size was calculated to be 228 subjects. Due to the lack of completed data and the impossibility of TSH measurement in some samples, the data of 216 subjects were finally analyzed. Written informed consent was obtained from all participants.

According to inclusion and exclusion criteria, 216 mothers aged 16–45 years were selected for the present study. The study protocol was explained to all participants. A random table number was used to randomly select the participants.

All questionnaires were asked by trained interviewers, and their information was entered into the software for analysis.

### 2.2. Inclusion Criteria

At least 1 year of living in Isfahan, no history of infertility, birth in Isfahan hospitals, and no active smoking before and during pregnancy are inclusion criteria.

Because we wanted to evaluate dietary intakes in the first trimester, so we only include those who were in their first trimester of pregnancy. In addition, the present study is a substudy of PERSIAN Birth Cohort. In the birth cohort study, several questionnaires were asked and blood samples were taken in the first trimester of pregnancy.

### 2.3. Exclusion Criteria

Major risk of small for gestational age (SGA) and intrauterine growth retardation (IUGR); medical complications including hypertension, diabetes, or kidney disease; cerclage until 24 weeks of pregnancy; and history of stillbirth or preterm labor are exclusion criteria. Those who were under thyroid disorder treatment, anemic, and malnourished mothers were excluded from the study. Salt restriction during pregnancy, maternal smoking, and antithyroid drug usage were considered exclusion criteria.

The IUGR is diagnosed when the estimated fetal weight is < 10% percentile for gestational age (GA) as calculated by biometric measurements.

### 2.4. DPI Calculation

A validated semiquantitative food frequency questionnaire (FFQ) with 90 items was used for the estimation of phytochemical intake [12]. A face-to-face questionnaire interview was conducted by trained interviewers. Data was collected in the first trimester of pregnancy. Grams of household-defined portion sizes of food items were calculated by Nutritionist IV software. The method proposed by McCarty was used to calculate DPI [14] by using the below formula:

 $$\begin{array}{c} \text{DPI}=\frac{\left[\text{daily energy derived from phytochemical}‐\text{rich foods }\left(\text{Kcal}\right)\right]}{\left[\text{total daily calorie intake }\left(\text{Kcal}\right)\right]}\times 100 \end{array}$$

Fruits, vegetables, legumes, whole grains, nuts, soy products, olives, and olive oil were used for the calculation of the DPI score.

### 2.5. Thyroid Function Measurement

A trained person obtained cord blood samples. Samples were centrifuged and were stored at −80°C. Chemiluminescence immunoassay with diagnostic kits of Bayer Company (Bayer Healthcare, Siemens, Berlin, Germany) was used to measure TSH and T4 concentrations (Vitros ECI; Ortho Clinical Diagnostics).

### 2.6. Statistical Analysis

Continuous quantitative and categorical variables were expressed as means ± SD or standard errors (SE) and numbers and percentages, respectively. Bell-shaped curve and Kolmogorov–Smirnov test were used to assess the normality of the data.

One-way analysis of variance (ANOVA) test was used to compare the means of continuous quantitative variables in different DPI quartiles. Analysis of covariance (ANCOVA) test was used to compare the mean of adjusted dietary intakes of macro- and micronutrients and food groups by age, gender, and total energy between different DPI quartiles. Frequencies of categorical variables among DPI quartiles were assessed by the Chi-square test. The correlation between DPI score (as continuous and quartiles) with LnTSH and FT4 was evaluated using multiple linear regression model adjusted with age, gender, educational level, income, physical activity (PA), GA, body mass index (BMI), and total energy. STATA 12.0 software (STATA Corp, College Station, Texas, USA) was used for all statistical analyses. Statistically significant was defined as *P* value less than 0.05.

## 3. Results

The data of 216 mothers aged 16–45 years old was analyzed. The mean (SD) age of mothers was 29.56 (5.50) years. Forty-seven percent of infants were girls. DPI scores in the first, second, third, and fourth quartiles were < 29.84, 29.84–37.25, 37.25–44.43, and > 44.43, and the mean DPIs were 25.03 ± 4.67, 33.87 ± 2.18, 40.64 ± 2.10, and 51.17 ± 4.98, respectively. The median (interquartile range (IQR)) for TSH and FT4 was 6.85 (4.58, 12.35) and 1 (0.90, 1.10), respectively.  Table 1 shows the characteristics of participants across various DPI quartiles. Participants in the higher DPI quartile in comparison with lower quartiles have higher BMI. However, differences in TSH, FT4, age, gender, and total energy according to quartiles of DPI quartiles were not significant (*P* > 0.05). Adjusted macro- and micronutrients and food groups across DPI quartiles are present in Table 2. Participants in Quartile 4 had higher adjusted means of protein (%), carbohydrate (%), fiber, iron, and magnesium and had higher intakes of whole grains and fruits in comparison with Quartile 1 (*P* < 0.05). Participants in Quartile 4 had lower adjusted mean fat (%) in comparison with Quartile 1 (*P* < 0.05).

Table 3 shows the results of regression analysis between DPI score (as continuous and quartiles) with LnTSH (TSH had no normal distribution, so the LnTSH was used in the analysis) and FT4 levels in crude and adjusted model with age, gender, educational level, income, PA, GA, energy intake, and BMI. There was not any significant relationship between the DPI score with LnTSH and FT4 in crude and adjusted models (*P* > 0.05). The adjusted mean FT4 for Quartile 2 was significantly more than Quartile 1 (Beta (SE) = 0.10 (0.04); *P* = 0.027).

## 4. Discussion

In the present study, the DPI score was not associated with neonatal TSH and FT4 levels. So, no significant association between neonatal thyroid function and maternal diet quality index was shown in the present study. In previous studies, no association was observed between diet and weight gain during pregnancy with cord blood TSH levels [15].

Maternal nutrition during pregnancy may impact endocrine patterns in the offspring. Several studies suggested that some dietary patterns and lifestyle were associated with the risk of chronic diseases; however, there is not sufficient information about the effects of the DPI on thyroid function, particularly among pregnant women. Neonatal TSH is a sensitive indicator that may be associated with nutritional status of pregnant women [7].

Evidence has shown that some noncommunicable diseases (NCDs) are correlated to utero and maternal factors that influence fetal metabolism and development. It can be considered the most important public health issue [16]. Maternal thyroid hormones especially during early pregnancy have an essential role in pre- and postnatal growth. Proper maternal and neonatal thyroid hormones are needed for neuropsychological development of the offspring [17].

Maternal thyroid hormones affect the development of the fetal brain until the production of thyroid hormone begins in the fetus at about 16–20 weeks of gestation. However, some nutrients such as iodine during whole pregnancy affect fetal thyroid function [18].

A normal diet and healthy diet are essential for a healthy pregnancy and optimal fetal and child development. A normal diet requires a balance of macronutrients (proteins, fats, carbohydrates, and fiber) and micronutrients (vitamins and trace elements). Deficiencies in micronutrients and macronutrients during pregnancy are correlated with poor pregnancy outcomes. These deficiencies enhance the risk of developing chronic disorders including endocrine disorders, insulin resistance, and metabolic dysfunction in children [19, 20]. Synthesis of thyroid hormones depends on sufficient dietary micronutrients. Several studies assessed the effects of single nutrients or foods during pregnancy on fetal development, thyroid hormone levels, and thyroid disturbances [19, 20]. Studies showed that iodine deficiency, soy products and cruciferous vegetable intake, selenium, zinc, vitamin D, and calcium levels can have an effect on thyroid function and thyroid hormone levels. The novel findings showed that deficiency of some nutrients during pregnancy led to sexually dimorphic changes particularly those related to thyroid function [21–23]. Unlike the evidence related to single nutrients, there is no definite information about the impact of specific dietary patterns on thyroid function especially during pregnancy. A study on an overweight and obese adult showed that the production of thyroid hormones slightly was inhibited by the Mediterranean diet, as an overall healthy diet. However, thyroid function did not change significantly [7].

Investigation of overall diet can show better diet-disease associations. The specific consequences of maternal DPI on neonatal thyroid function have not previously been investigated [19]. An animal study showed that a maternal high-fat diet could change the fetal thyroid levels in the third trimester [24]. Maternal high-fat diets change the hepatic histone code and transcriptional and epigenetic of the fetal thyroid axis. Thus, exposure to a high-fat diet in utero may disturb the fetal thyroid homeostasis [25]. Unhealthy dietary patterns with high fat lead to hyperleptinaemia that is correlated with high circulating thyroid hormones [26].

Plant-based diets contain fresh fruits, legumes, and vegetables and they include several useful nutrients such as vitamins, minerals, antioxidants, phytochemicals, and fiber. Plant foods may reduce chronic diseases and endocrine disorders. Diets rich in fruit, vegetables, and legumes that contain complex carbohydrates are fermented by healthy gut microbiota. They produce short-chain fatty including butyrate, propionate, and acetate that are beneficial for human health, including thyroid function [27, 28].

In addition, fatty red meats, processed meats, processed foods, and heavily salted and pickled foods may increase endocrine disorders. Phytochemicals can ameliorate the adverse effects of unhealthy dietary patterns [3].

Phytochemicals have antioxidant and anti-inflammatory properties, and they have beneficial effects on health. They influence on cell cycle regulation and hormones. DPI is a quantitative index for assessing the effects of phytochemicals on health [29, 30]. It may have high practical and clinical use and can provide important information about diet [31].

Foods rich in phytochemicals decrease oxidative stress significantly. Phytochemicals are mainly found in fruits, vegetables, cereals, and tea. Several studies indicated that DPI was inversely associated with several NCDs [30, 31]. These findings showed that diets with a high proportion of phytochemical-rich plant foods may affect health status. Significantly decreased levels of antioxidants were reported in thyroid dysfunction and thyroid cancer [32–34].

The present study was done in a nationally representative sample of pregnant women that increases the generalizability of our findings. In addition, dietary intake was collected by train questionnaire, and the data was not based on self-report data.

Maternal thyroid function may affect neonatal thyroid function. We suggest that the level of maternal thyroid hormones, autoantibody levels, and maternal iodine status can be evaluated in future studies. More studies with larger sample sizes are needed to find more results related to nutrition and thyroid hormones.

Our study has some advantages. First, data of a population-based prospective cohort study was used to evaluate the correlation between maternal DPI and levels of neonatal thyroid hormone, and it might provide clues to establish causality. Second, no studies have assessed the effect of maternal DPI on neonatal thyroid function.

Our study also had a limitation. We did not measure urinary iodine concentration, which may be related to thyroid function. In addition, potential unmeasured confounders might affect results.

## 5. Conclusion

The present study reports no significant relationship between maternal DPI with neonatal TSH and FT4 levels. The amount of foods rich in phytochemical compound consumption can be important, and their beneficial effects may be shown with high consumption. There is little information about the impact of maternal DPI on fetal and neonatal thyroid function. It highlights the need for further studies to investigate the impacts of maternal quality of diet on neonatal thyroid dysfunction.

## Figures and Tables

**Table 1:** Characteristics of participants in the study across quartiles of dietary phytochemical index.

	**Total**		**Q1 (** **n** = 54**)**		**Q2 (** **n** = 54**)**		**Q3 (** **n** = 54**)**		**Q4 (** **n** = 54**)**		**P**
	**Mean**	**SD**	**Mean**	**SD**	**Mean**	**SD**	**Mean**	**SD**	**Mean**	**SD**	
DPI	37.68	10.27	25.03	4.67	33.87	2.18	40.64	2.10	51.17	4.98	<0.001
Age	29.56	5.50	28.85	5.14	29.43	5.58	30.07	5.61	29.87	5.74	0.272
BMI	25.07	4.96	23.25	4.37	25.50	6.04	25.31	3.21	26.18	5.54	0.010
Energy	571.56	284.23	562.74	198.43	587.56	443.71	547.20	189.06	588.72	233.41	0.525
Gestational age (week)	38.86	1.06	38.98	1.08	38.83	1.07	38.83	1.11	38.80	1.00	0.688
Total physical activity (MET–min/week)	1143.88	1092.97	1066.29	1058.75	1276.64	1215.48	1158.25	1265.19	1073.68	800.65	0.296
TSH median (IQR)	9.08	6.85	9.86	7.30	10.08	8.64	7.06	3.31	9.33	6.77	0.399
	6.85	(4.58, 12.35)	7.85	(5.05, 10.40)	6.55	(5.05, 10.73)	6.25	(4.30, 8.55)	7.75	(4.40, 12.35)	
FT4 median (IQR)	1.01	0.19	1.00	0.17	1.04	0.20	1.00	0.21	0.99	0.17	0.525
	1.00	(0.90, 1.10)	1.00	(0.90, 1.10)	1.00	(0.90, 1.18)	1.05	(0.90, 1.20)	1.00	(0.90, 1.10)	
Gender	*n*	%	*n*	%	*n*	%	*n*	%	*n*	%	0.192
Girls	102	47%	25	24.5%	32	31.4%	24	23.5%	21	20.6%	
Boys	113	53%	29	25.7%	22	19.5%	29	25.7%	33	29.2%	
Income											
Low	45	19.6	15.00	0.34	12.00	0.27	9	20.5%	8	18.2%	0.55
Moderate	161	70.0	36.00	0.24	36.00	0.24	39	25.8%	40	26.5%	
High	10	4.3	1.00	0.10	3.00	0.30	2	20.0%	4	40.0%	
Education											
No or primary	15	6.5	6	0.46	3	0.23	3	23.1%	1	7.7%	0.34
Secondary less than 12 years	122	53.0	27.00	0.23	25.00	0.22	31	26.7%	33	28.4%	
Bachelors	73	31.7	16.00	0.23	22.00	0.31	16	22.9%	16	22.9%	
Master or Doctorate	6	2.6	3.00	0.50	1.00	0.17	0	0.0%	2	33.3%	

Abbreviations: BMI, body mass index; DPI, dietary phytochemical index; FT4, free thyroxine; IQR, interquartile range; TSH, thyroid-stimulating hormone.

**Table 2:** Adjusted maternal dietary intakes in the first trimester of pregnancy across quartiles of dietary phytochemical index.

	**Q1**		**Q2**		**Q3**		**Q4**		**P**
	**Mean**	**SE**	**Mean**	**SE**	**Mean**	**SE**	**Mean**	**SE**	
Protein (%)	0.11	0.002	0.11	0.002	0.11	0.002	0.12	0.002	0.009
Carbohydrate (%)	0.57	0.01	0.59	0.01	0.61	0.01	0.64	0.01	< 0.001
Fat (%)	0.34	0.01	0.32	0.01	0.31	0.01	0.27	0.01	< 0.001
Total fiber	5.38	0.23	6.49	0.23	7.18	0.23	8.59	0.23	< 0.001
Calcium	202.92	9.78	230.32	9.92	227.50	9.89	215.30	9.99	0.184
Iron	2.23	0.06	2.39	0.06	2.51	0.06	2.78	0.06	< 0.001
Magnesium	67.20	1.39	74.68	1.41	77.30	1.41	79.44	1.42	< 0.001
Sodium	836.04	36.40	946.40	36.94	851.36	36.82	932.83	37.18	0.077
Zinc	1.87	0.04	1.95	0.04	2.01	0.04	1.99	0.04	0.075
Selenium	11.50	0.53	11.56	0.53	11.64	0.53	11.68	0.54	0.996
Whole grains	13.37	2.58	19.86	2.62	27.69	2.61	42.82	2.63	< 0.001
Legumes	7.21	0.84	6.37	0.85	8.50	0.85	9.28	0.86	0.079
Vegetables	83.17	5.67	98.53	5.75	87.41	5.73	89.41	5.79	0.282
Fruits	93.31	11.72	139.86	11.90	167.03	11.86	186.69	11.97	< 0.001
Nuts	1.74	0.37	2.63	0.37	2.63	0.37	3.03	0.38	0.094

**Table 3:** Association between DPI and cord blood thyroid hormones.

	**LnTSH**			**FT4**		
	**Beta**	**SE**	**P**	**Beta**	**SE**	**P**
DPI						
Crude	−0.04	0.44	0.92	−0.02	0.13	0.888
Adjusted^∗^	0.41	0.51	0.417	−0.16	0.16	0.319
DPI quartiles						
Q1	Ref.			Ref.		
Q2	−0.05	0.16	0.749	0.10	0.04	0.027
Q3	−0.11	0.15	0.478	0.05	0.04	0.251
Q4	0.07	0.15	0.624	0.04	0.04	0.332

Abbreviations: DPI, dietary phytochemical index; FT4, free thyroxine; TSH, thyroid-stimulating hormone.

^*^Adjusted with age, gender, educational level, income, physical activity, gestational age, BMI, and total energy.

## Data Availability

The data that support the findings of this study are available from the corresponding authors, upon reasonable request.

## References

[1] Mulder T. A., van den Dries M. A., Korevaar T. I. M., Ferguson K. K., Peeters R. P., Tiemeier H. (2019). Organophosphate pesticides exposure in pregnant women and maternal and cord blood thyroid hormone concentrations. *Environment International*.

[2] Eng L., Lam L. (2020). Thyroid function during the fetal and neonatal periods. *NeoReviews*.

[3] Adedapo K., Ogunwale K., Musa A., Olufemi O. (2014). Dietary pattern and antioxidants levels in patients with simple goiter and thyroid cancer. *International Journal of TROPICAL DISEASE & Health*.

[4] Chen S., Peng Y., Zhang H., Zou Y. (2023). Relationship between thyroid function and dietary inflammatory index in Hashimoto thyroiditis patients. *Medicine (Baltimore)*.

[5] Fontana L., Klein S., Holloszy J. O., Premachandra B. N. (2006). Effect of long-term calorie restriction with adequate protein and micronutrients on thyroid hormones. *The Journal of Clinical Endocrinology & Metabolism*.

[6] Trumpff C., Vandevijvere S., Moreno-Reyes R. (2015). Neonatal thyroid-stimulating hormone level is influenced by neonatal, maternal, and pregnancy factors. *Nutrition Research*.

[7] Zupo R., Castellana F., Panza F. (2020). Adherence to a mediterranean diet and thyroid function in obesity: a cross-sectional Apulian survey. *Nutrients*.

[8] Kouidrat Y., Diouf M., Desailloud R., Louhou R. (2019). Effects of a diet plus exercise program on thyroid function in patients with obesity. *Metabolism Open*.

[9] Eslami O., Khoshgoo M., Shidfar F. (2020). Dietary phytochemical index and overweight/obesity in children: a cross-sectional study. *BMC Research Notes*.

[10] Azizi-Soleiman F., Khoshhali M., Heidari-Beni M., Qorbani M., Ali Pourmirzaei M., Kelishadi R. (2021). Higher dietary phytochemical index is associated with anthropometric indices in children and adolescents: the weight disorders survey of the CASPIAN-IV study. *International Journal for Vitamin and Nutrition Research*.

[11] Tan K. M., Chu A. H., Loy S. L. (2021). Association of cord blood thyroid-stimulating hormone levels with maternal, delivery and infant factors. *Annals of the Academy of Medicine, Singapore*.

[12] Zare Sakhvidi M. J., Danaei N., Dadvand P. (2021). The prospective epidemiological research studies in IrAN (PERSIAN) birth cohort protocol: rationale, design and methodology. *Longitudinal and Life Course Studies*.

[13] Berg V., Nøst T. H., Skeie G. (2017). Thyroid homeostasis in mother-child pairs in relation to maternal iodine status: the MISA study. *European Journal of Clinical Nutrition*.

[14] McCarty M. F. (2004). Proposal for a dietary “phytochemical index”. *Medical Hypotheses*.

[15] Herbstman J., Apelberg B. J., Witter F. R., Panny S., Goldman L. R. (2008). Maternal, infant, and delivery factors associated with neonatal thyroid hormone status. *Thyroid*.

[16] Lain S. J., Rifas-Shiman S. L., Pearce E. N., Nassar N., Oken E. (2020). Neonatal thyroxine, maternal thyroid function, and cognition in mid-childhood in a US cohort. *Maternal and Child Health Journal*.

[17] Huget-Penner S., Feig D. S. (2020). Maternal thyroid disease and its effects on the fetus and perinatal outcomes. *Prenatal Diagnosis*.

[18] Abel M. H., Korevaar T. I., Erlund I. (2018). Iodine intake is associated with thyroid function in mild to moderately iodine deficient pregnant women. *Thyroid*.

[19] Hofstee P., McKeating D. R., Bartho L. A., Anderson S. T., Perkins A. V., Cuffe J. S. (2020). Maternal selenium deficiency in mice alters offspring glucose metabolism and thyroid status in a sexually dimorphic manner. *Nutrients*.

[20] Hofstee P., McKeating D. R., Perkins A. V., Cuffe J. S. (2018). Placental adaptations to micronutrient dysregulation in the programming of chronic disease. *Clinical and Experimental Pharmacology and Physiology*.

[21] Hsu C.-N., Tain Y.-L. (2019). The good, the bad, and the ugly of pregnancy nutrients and developmental programming of adult disease. *Nutrients*.

[22] Habibi N., Grieger J. A., Bianco-Miotto T. (2020). A review of the potential interaction of selenium and iodine on placental and child health. *Nutrients*.

[23] Mackawy A. M. H., Al-Ayed B. M., Al-Rashidi B. M. (2013). Vitamin D deficiency and its association with thyroid disease. *International Journal of Health Sciences*.

[24] Suter M. A., Sangi-Haghpeykar H., Showalter L. (2012). Maternal high-fat diet modulates the fetal thyroid axis and thyroid gene expression in a nonhuman primate model. *Molecular Endocrinology*.

[25] Sharma R., Bharti S., Kumar K. H. (2014). Diet and thyroid-myths and facts. *Journal of Medical Nutrition and Nutraceuticals*.

[26] Franco J. G., Fernandes T. P., Rocha C. P. (2012). Maternal high-fat diet induces obesity and adrenal and thyroid dysfunction in male rat offspring at weaning. *The Journal of Physiology*.

[27] Yahia E. M., García-Solís P., Celis M. E. M. (2019). *Contribution of fruits and vegetables to human nutrition and health*.

[28] Tomova A., Bukovsky I., Rembert E. (2019). The effects of vegetarian and vegan diets on gut microbiota. *Frontiers in Nutrition*.

[29] Carnauba R. A., Chaves D. F., Baptistella A. B., Paschoal V., Naves A., Buehler A. M. (2017). Association between high consumption of phytochemical-rich foods and anthropometric measures: a systematic review. *International Journal of Food Sciences and Nutrition*.

[30] Kim M., Park K. (2020). Association between phytochemical index and metabolic syndrome. *Nutrition Research and Practice*.

[31] Farhangi M. A., Najafi M., Jafarabadi M. A., Jahangiry L. (2017). Mediterranean dietary quality index and dietary phytochemical index among patients candidate for coronary artery bypass grafting (CABG) surgery. *BMC Cardiovascular Disorders*.

[32] Im J., Kim M., Park K. (2020). Association between the phytochemical index and lower prevalence of obesity/abdominal obesity in Korean adults. *Nutrients*.

[33] McEwen B., Bingham M. (2019). Vegan diet and chronic disease: a brief report. *Journal of the Australian Traditional-Medicine Society*.

[34] Dreher M. L. (2018). *Dietary Patterns and Whole Plant Foods in Aging and Disease*.

